# Comparison of RT-qPCR and RT-ddPCR with Rift valley fever virus (RVFV) RNA

**DOI:** 10.1038/s41598-023-29023-y

**Published:** 2023-02-22

**Authors:** Changwoo Park, Dongju Park, Zohaib Ul Hassan, Sang Ho Choi, Seil Kim

**Affiliations:** 1 Microbiological Analysis Team, Group for Biometrology, Korea Research Institute of Standards and Science, Daejeon, 34113 Republic of Korea; 2 Convergent Research Center for Emerging Virus Infection, Korea Research Institute of Chemical Technology, Daejeon, 34114 Republic of Korea; 3 Department of Agricultural Biotechnology, Seoul National University, Seoul, 08826 Republic of Korea; 4 Theragen Bio Co., Ltd., Seoul, 16229 Republic of Korea; 5 Department of Bio-Analysis Science, University of Science & Technology, Daejeon, 34113 Republic of Korea; 6 Center for Food and Bioconvergence, Seoul National University, Seoul, 08826 Republic of Korea

**Keywords:** Microbiology, Molecular biology

## Abstract

Rift valley fever (RVF) is an important zoonotic disease caused by the Rift valley fever virus (RVFV) which can affect ruminants and humans. In this study, a comparison was done of the reverse transcription-quantitative polymerase chain reaction (RT-qPCR) and reverse transcription-droplet digital PCR (RT-ddPCR) assays with synthesized RVFV RNA, cultured viral RNA, and mock clinical RVFV RNA samples. The genomic segments (L, M, and S) of three RVFV strains (BIME01, Kenya56, and ZH548) were synthesized and used as templates for in vitro transcription (IVT). Both the RT-qPCR and RT-ddPCR assays for RVFV did not react with any of the negative reference viral genomes. Thus, both the RT-qPCR and RT-ddPCR assays are specific to RVFV. The comparison of both the RT-qPCR and RT-ddPCR assays with serially diluted templates showed that the LoD of both assays are similar, and a concordant of the results was observed. The LoD of both assays reached the practical measurable minimum concentration. Taken altogether, the sensitivity of the RT-qPCR and RT-ddPCR assays is similar, and the material measured by RT-ddPCR can be used as a reference material for RT-qPCR.

## Introduction

Rift valley fever was first identified in the Rift Valley of East Africa and isolated in 1930^1^. This acute fever is caused by the rift valley fever virus (RVFV), which can affect humans with mild to severe symptoms. Additionally, this virus is pathogenic to livestock and wildlife, and sheep are the most susceptible to it^2^. New-born lambs may die within hours after the onset of symptoms, and most do not survive. This virus belongs to the family Bunyaviridae and causes mild symptoms such as fever and muscle pain and severe symptoms such as loss of sight and severe headaches and confusion^3^. The mortality rates of humans infected with this virus have been reported to be from 0.5 to 2%^4^. However, humans can have complications such as nausea, vomiting, diarrhea, hemorrhage, and jaundice^1^. It is spread by touching the blood of an infected animal, breathing the air around an infected animal being slaughtered, drinking the raw milk of an infected animal, or being bitten by an infected mosquito.

RVFV is a single-stranded RNA virus and consists of three segments: large (L), medium (M), and small (S)^5^. The L segment is the longest segment (6.4 kb) and encodes the RNA-dependent RNA polymerase (RdRp); the M segment spans 3.2 kb and encodes surface glycoproteins (Gn and Gc) and the non-structural protein NSm. The S segment is the shortest (1.7 kb) and encodes nucleoprotein N and non-structural protein NSs^6–8^. The major virulence factor of RVFV is NSs, and it interferes with the host immune response by inhibiting mRNA synthesis^9,10^. In addition, NSs can shut down the transcription of the interferon (IFN) production system, which is an important factor for the immune system to fight off foreign virus infections^11,12^.

For the past several decades, RT-qPCR has been the gold standard method of diagnostics for viruses such as SARS-CoV-2 and RVFV^13–16^. Because the RT-qPCR methods are well-established and can be developed with partial genome sequence information, they are used as initial diagnostic methods for newly emerging viruses such as SARS-CoV-2^17–19^. In contrast to the relative quantification of RT-qPCR, reverse-transcription-droplet digital PCR (RT-ddPCR) is a powerful method for absolute quantification^17,20–23^. Even though several procedures including making the droplets, traditional PCR, and droplet reading are required, this assay can perform quantification without a standard curve compared to RT-qPCR^24^.

In this study, the genome segments (L, M, and S) of three RVFV strains (BIME01, Kenya56, and ZH548) were synthesized. Additionally, synthesized viral genomic DNAs were used as templates for in vitro transcription (IVT) of the viral RNA. The IVT RNAs and eleven negative viral RNAs were used as templates for RT-qPCR and RT-ddPCR, and the results showed that both assays are specific to RVFV. The specificity, the LoD (Limit of Detection), and the LoQ (Limit of Quantification) of both assays were determined with RVFV viral and mock patient RNA. Our results show that there is no significant difference in the LoD between the well-optimized RT-qPCR and RT-ddPCR assays. Because both methods are based on PCR and the primer–probe sets are compatible, the materials in the absolute quantification by RT-ddPCR can be used as references for RT-qPCR.

## Results

### Screening and validation of the primer–probe sets

The sequences of the primer–probe sets were aligned with various RVFV genome sequences. Although there were one or two mismatches with some genomic sequences, the RVFV L, M, and S primer–probe sets were used to quantify the synthetic genomes of the BIME01, Kenya56, and ZH548 strains in this study. The alignment results indicate that the primer–probe set can cover not only the lineages A, D, and K but also cover the lineages B, C, E, F, and G. However, the BeAn24262 strain did not align well with the other strains and used primer–probe sets. In addition, the sequences of the RVFV S primer–probe sets showed multiple mismatches with the genome sequences of the OV_35/74 and 688/78 strains, indicating that the primer–probe is not applicable to those strains.

Among the various published primer–probe sets, the primer–probe sets used in this study were the most conserved in the RVFV genome sequences^25–29^. There were more conservative primer–probe sets, but those sets contain degenerate nucleotides^30^. Eleven RNAs from the reference viruses were quantified by Quantus fluorometer, RT-qPCR and RT-ddPCR. The concentrations of the reference viral RNA are listed in Supplementary Table S1. In the results for the RT-qPCR, all negative reference viral RNAs were not positive for the RVFV L, M, and S primer–probe sets. In the RT-ddPCR result, all negative reference viral RNAs showed a signal of less than 1 copy number/μl (Supplementary Table S2) while each dedicated primer–probe set for each negative reference confirmed the presence of the RNA. These results confirm that the primer–probe sets used in this study are specific to the RVFV for both the RT-qPCR and RT-ddPCR assays.

### Measurement of synthetic RVFV RNA using RT-qPCR and RT-ddPCR

The size distribution of the RVFV IVT RNA was initially determined using a Bioanalyzer. The concentrations of RNA are shown in Supplementary Table S3. The sizes of the synthesized RNA of the L, M, and S segments were 6, 4 and 2 kb, respectively.

**Figure 1:**
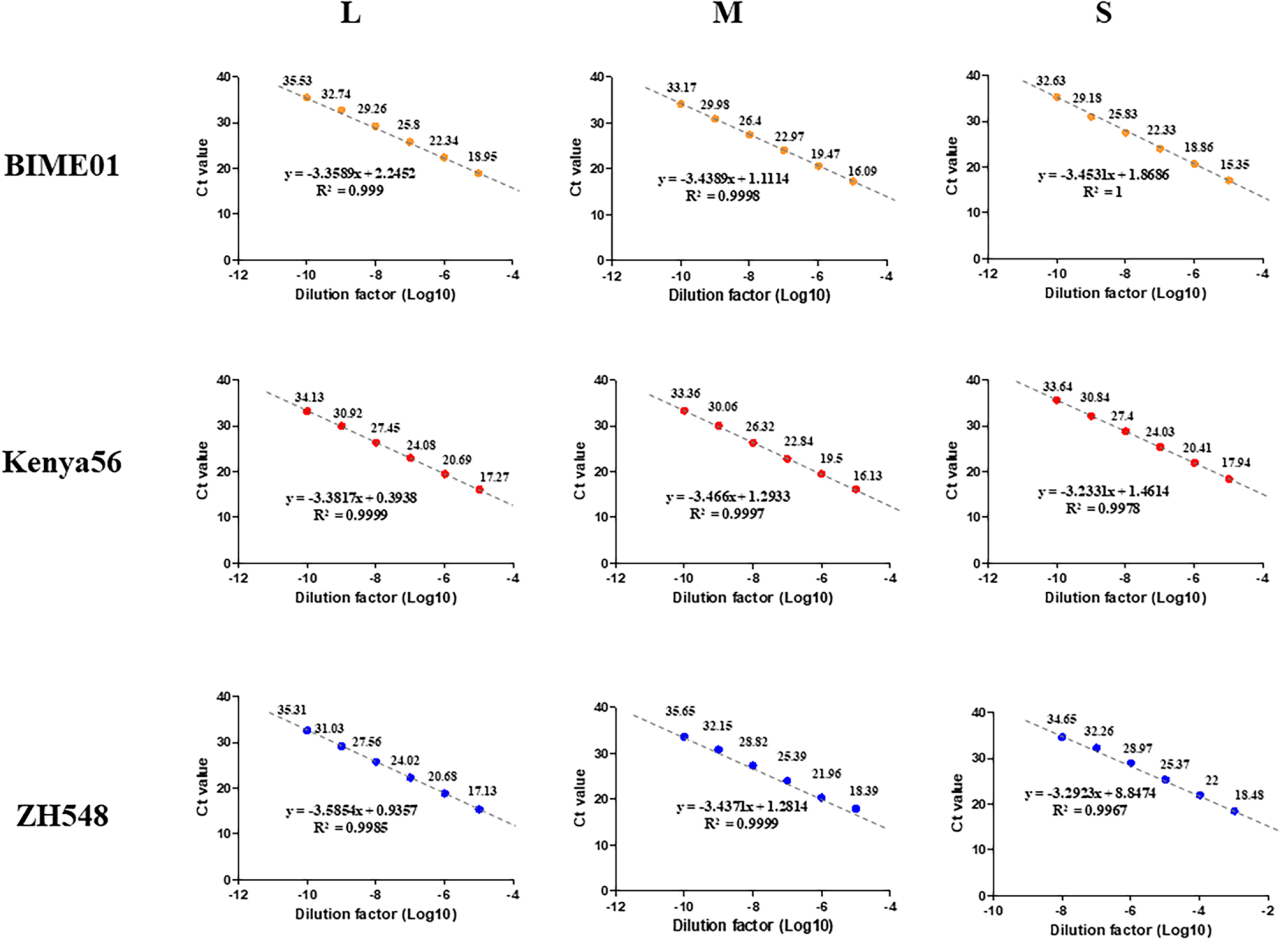
The Ct value results of the RT-qPCR. The colors orange, red, and blue indicate the BIME01, Kenya56, and ZH548 strain of the Rift valley fever virus, respectively. Each strain contains the L, M, and S segments. The number above the dot indicates the mean Ct value.

**Figure 2:**
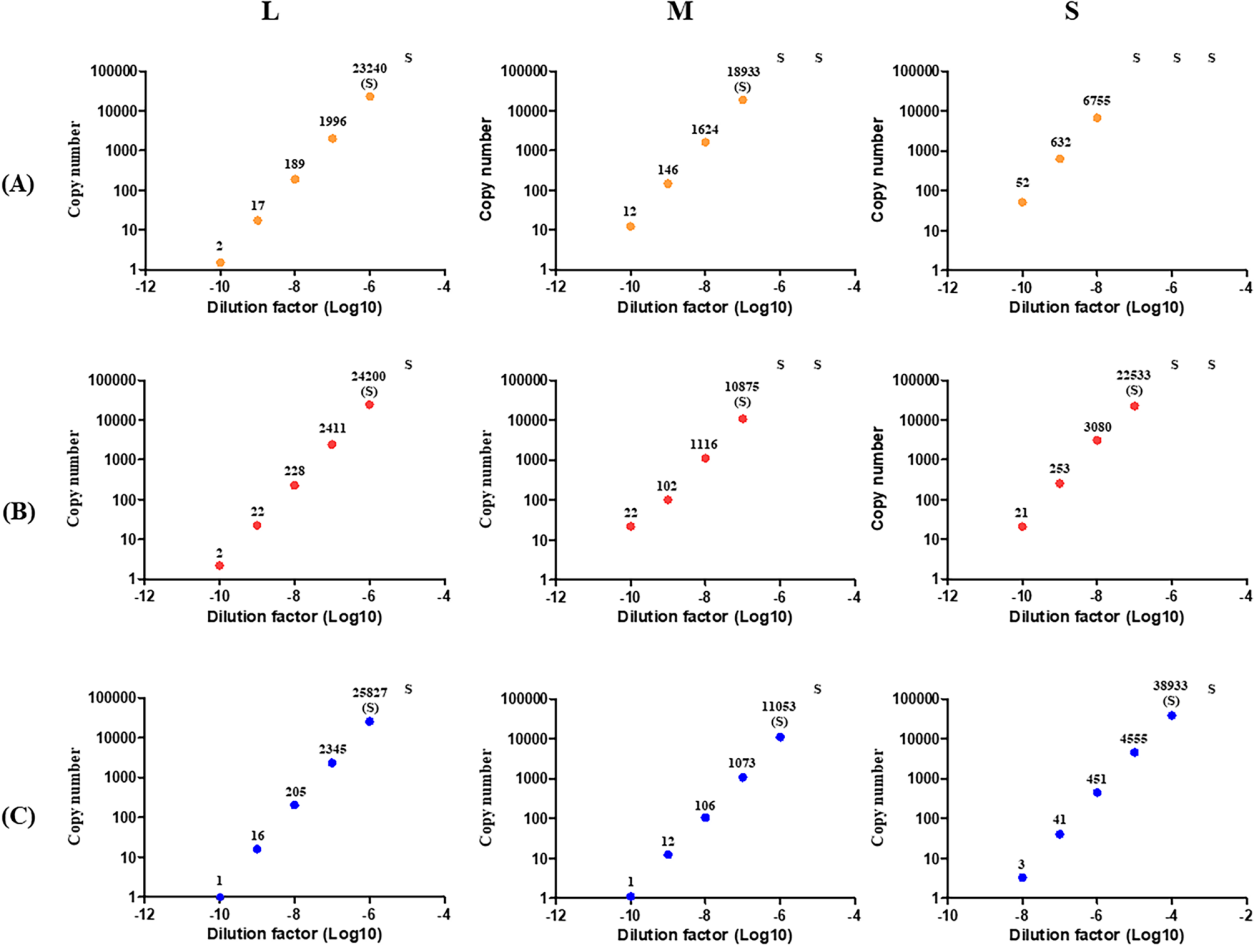
The copy number results of the RT-ddPCR. The (**A**), (**B**), and (**C**) indicate the BIME01, Kenya56, and ZH548 strains of the Rift valley fever virus, respectively. Each strain contains the L, M, and S segments. The number above the dot indicates average copy number and ‘S’ indicates saturation.

The IVT RNAs were serially diluted (tenfold) and used as templates for the RT-qPCR with each primer–probe set (Fig. 1). The resulting Ct values are listed in Supplementary Table S4. The range of the Ct values was from 15.35 to 35.65. The regression coefficients (R^2^) of the RVFV BIME01, Kenya56, and ZH548 were greater than 0.9967, and all results showed linear standard curves. The PCR efficiency of the assays were from 90.07 to 103.85%. The same RVFV IVT was also used as templates for RT-ddPCR (Fig. 2 and Supplementary Table S5). The copy numbers of the serially diluted templates showed linearity. All the RVFV IVT samples were measured from diluted 10^3^ to 10^10^ templates (Supplementary Tables S4 and S5). The RT-ddPCR results showed that the LoQ of the assays was approximately ten or less copies. In L segment, the LoQ average of three strains showed 1.7 copy number/μl. In M segment, the LoQ average of three strains showed 0.3 copy number/μl. In S segment, the LoQ average of three strains showed 3 copy number/μl.

Considering these RT-ddPCR results and the standard curves of the RT-qPCR, Ct values of more than approximately 37 should be regarded as negative. These values indicated that the LoQ of both the RT-qPCR and RT-ddPCR had a copy number of approximately 1–2 per μl. Regardless of the assays and template sequences, the copy number from the RT-ddPCR and the Ct value from RT-qPCR were well-matched; when the Ct value from RT-qPCR is approximately 34 or higher, the copy number from RT-ddPCR is approximately ten or less. However, considering most RT-qPCR assays use cutoff values of 35–40, a Ct value of 1–2 copies/μl can be regarded as very marginal or negative.

### LoQ assessment using cultured and mock clinical RNA

Cultured and mock clinical RVFV RNAs were used to assess the LoQ of RT-qPCR and RT-ddPCR^31^. The Ct values of the cultured viral RNA showed linearity with the serially diluted samples (Table 1a). All R^2^ values from the linear regression of the RT-qPCR results were greater than 0.999. The PSD of all the RT-qPCR results with the cultured viral RNA was less than 0.48. The copy numbers of the same serially diluted cultured viral RNA were determined using RT-ddPCR, and the linear regression of the results also showed linearity (Table 1b). The relative standard deviations of the copy number with the cultured viral RNA were approximately 10% except for templates at very low concentrations.

**Table 1:** Limit of Quantification (LoQ) of the cultured RVFV RNA by RT-qPCR with the L, M and S segment assay.

Dilution rate	Primer-probes	(a) Ct value of cultured RNA														
		RVFV L					RVFV M					RVFV S				
	Run	Run 1	Run 2	Run 3	Mean	RSD (%)	Run 1	Run 2	Run 3	Mean	RSD (%)	Run 1	Run 2	Run 3	Mean	RSD (%)
NTC		U	U	U	U	0	U	U	U	U	0	U	U	38.33	38.33	0
Undiluted		22.2 ± 0.05	22.28 ± 0.05	22.27 ± 0.02	22.25 ± 0.04	0.2	23.68 ± 0.23	23.53 ± 0.2	23.48 ± 0.2	23.56 ± 0.21	0.9	21.93 ± 0.02	21.98 ± 0.03	21.86 ± 0.08	21.92 ± 0.05	0.2
10^1^		26.63 ± 0.08	25.79 ± 0.03	25.72 ± 0.04	26.05 ± 0.06	0.2	28.4 ± 0.16	27.13 ± 0.2	26.92 ± 0.14	27.48 ± 0.17	0.6	24.74 ± 0.06	25.65 ± 0.02	25.49 ± 0.01	25.29 ± 0.04	0.1
10^2^		30.11 ± 0.12	29.37 ± 0.06	29.56 ± 0.08	29.68 ± 0.09	0.3	32.16 ± 0.32	30.87 ± 0.06	30.89 ± 0.19	31.3 ± 0.21	0.7	28.39 ± 0.05	29.39 ± 0.02	29.53 ± 0.1	29.11 ± 0.07	0.2
10^3^		33.61 ± 0.18	32.79 ± 0.31	32.88 ± 0.27	33.09 ± 0.26	0.8	35.51 ± 0.06	34.22 ± 0.19	34.24 ± 0.26	34.66 ± 0.19	0.5	32.02 ± 0.18	32.82 ± 0.1	32.87 ± 0.07	32.57 ± 0.13	0.4
10^4^		36.8 ± 0.18^†^	36.89 ± 0.04	36.97 ± 0.81	36.9 ± 0.48	1.3	39.34 ± 0.22	38.17 ± 0.32	38.44 ± 0.42	38.65 ± 0.33	0.9	35.51 ± 0.45	36.78 ± 0.24	36.74 ± 0.37	36.34 ± 0.36	1
10^5^		U	U	U	U	0	U	39.52*	U	39.52	0	38.44 ± 1.61	U	U	38.44 ± 1.61	0

Dilution rate	Primer-probes	(b) Cultured RVFV RNA copy number (copy/μl)														
		RVFV L					RVFV M					RVFV S				
	Run	Run 1	Run 2	Run 3	Mean	RSD (%)	Run 1	Run 2	Run 3	Mean	RSD (%)	Run 1	Run 2	Run 3	Mean	RSD (%)
NTC		0	0	0	0	0	0	0	0	0	0	0	0	0.24	0.07	0
Undiluted		9417 ± 92	9403 ± 496	9552 ± 371	9457 ± 362	3.8	37,200 ± 3487	36,707 ± 4141	36,133 ± 2053	36,680 ± 3343	9.1	39,653 ± 7638	40,533 ± 4772	40,800 ± 6729	40,329 ± 6491	16.1
10^1^		931 ± 15	967 ± 13	905 ± 13	934 ± 14	1.5	3547 ± 148	3620 ± 146	3395 ± 10	3520 ± 120	3.4	4676 ± 156	4917 ± 24	4285 ± 184	4626 ± 140	3
10^2^		69 ± 2	73 ± 3	63 ± 4	68 ± 3.36	4.9	219 ± 6.6	235 ± 9.12	200 ± 1.83	218 ± 6.59	3	327 ± 14	347 ± 14	288 ± 14	321 ± 14	4.4
10^3^		5.75 ± 0.45	7.31 ± 2.09	7.47 ± 1.01	6.84 ± 1.36	19.9	21 ± 1.22	20 ± 0.46	21 ± 3.03	21 ± 1.9	9.1	29 ± 2.27	33 ± 0.23	30 ± 1.67	31 ± 1.63	5.3
10^4^		0.56 ± 0.14	0.87 ± 0.08	0.8 ± 0.04	0.74 ± 0.1	13	1.31 ± 0.27	1.6 ± 0.24	1.24 ± 0.39	1.38 ± 0.31	22.3	2.65 ± 0.18	2.41 ± 0.31	2.48 ± 1.5	2.52 ± 0.89	35.3
10^5^		0	0	0	0	0	0.16 ± 0.14^†^	0.17 ± 0.3*	0.36 ± 0.14	0.23 ± 0.21	89.6	0.25 ± 0.24^†^	0.39 ± 0.19	0.27 ± 0.46*	0.3 ± 0.32	105.9

(a) Each run represents the mean Ct value of three replicates within an independent run.

*Only one of three replicates were positive.

^†^Only two of three replicates were positive.

The ‘U’ indicates underdetermined.

(b) Each run represents the mean copy number of three replicates within an independent run.

The mock clinical RNA was also measured in the same manner as the viral RNA measurement. Both the Ct value and copy number of the serially diluted mock clinical samples showed linearity (Table 2a,b). The R^2^ values of the mock clinical samples were greater than 0.999. The PSD of all the RT-qPCR results with the mock clinical samples was less than 1.13. The results for the cultured RNA indicate that the LoQ values of the L, M, and S segments for both assays were 0.74, 0.23, and 0.3 copies/µL, respectively. For the mock clinical RNA, the LoQ values of the L, M, and S segments for both assays were 0.06, 0.16, and 0.46 copies/µL, respectively.

**Table 2:** Limit of Quantification (LoQ) of the RVFV mock clinical RNA by RT-qPCR with the L, M and S segment assay.

Dilution rate	Primer-probes	(a) Ct value of mock clinical RNA														
		RVFV L					RVFV M					RVFV S				
	Independent Run	Run 1	Run 2	Run 3	Mean	RSD (%)	Run 1	Run 2	Run 3	Mean	RSD (%)	Run 1	Run 2	Run 3	Mean	RSD (%)
NTC		U	U	U	U	0	U	U	U	U	0	U	U	U	U	0
Undiluted		22.45 ± 0.02	22.32 ± 0.05	22.71 ± 0.05	22.49 ± 0.04	0.2	23.21 ± 0.04	23.22 ± 0.1	23.27 ± 0.19	23.23 ± 0.11	0.5	21.18 ± 0.05	21.18 ± 0.1	21.1 ± 0.16	21.15 ± 0.11	0.5
10^1^		25.79 ± 0.03	25.79 ± 0.1	26.19 ± 0.13	25.92 ± 0.1	0.4	26.86 ± 0.1	26.64 ± 0.11	26.61 ± 0.13	26.7 ± 0.11	0.4	24.64 ± 0.05	24.66 ± 0.05	24.72 ± 0.04	24.68 ± 0.05	0.2
10^2^		29.34 ± 0.08	29.32 ± 0.19	29.64 ± 0.05	29.43 ± 0.12	0.4	30.26 ± 0.14	30.44 ± 0.23	29.77 ± 0.02	30.16 ± 0.15	0.5	28.37 ± 0.09	28.27 ± 0.1	28.21 ± 0.09	28.28 ± 0.1	0.3
10^3^		32.67 ± 0.22	32.96 ± 0.45	33.02 ± 0.23	32.88 ± 0.32	1	33.71 ± 0.24	33.98 ± 0.13	33.43 ± 0.19	33.71 ± 0.19	0.6	31.79 ± 0.15	31.75 ± 0.15	31.84 ± 0.11	31.79 ± 0.14	0.4
10^4^		37.13 ± 1.52	37.07 ± 1.08	36.65 ± 0.6	36.95 ± 1.13	3.1	38.28 ± 0.91	38.2 ± 0.89	38.6 ± 0.9	38.35 ± 0.9	2.3	35.46 ± 0.72	35.87 ± 0.85	35.71 ± 0.25	35.68 ± 0.66	1.8
10^5^		37.48 ± 0.78^†^	38.28*	U	37.74	0	U	U	U	U	0	38.04 ± 0.11	37.84 ± 0.43	37.52 ± 1.69	37.8 ± 1.01	2.7

Dilution rate	Primer-probes	(b) Mock clinical RNA copy number (copy/μl)														
		RVFV L					RVFV M					RVFV S				
	Independent Run	Run 1	Run 2	Run 3	Mean	RSD (%)	Run 1	Run 2	Run 3	Mean	RSD (%)	Run 1	Run 2	Run 3	Mean	RSD (%)
NTC		0	0	0	0	0	0	0	0	0	0	0	0	0	0	0
Undiluted		9224 ± 203	9135 ± 190	9269 ± 574	9209 ± 368	4	31,760 ± 5897	31,893 ± 3121	31,733 ± 3972	31,796 ± 4483	14.1	35,320 ± 2226	40,533 ± 5829	45,467 ± 231	40,440 ± 3605	8.9
10^1^		931 ± 18	1001 ± 190	881 ± 20	938 ± 111	11.8	3401 ± 177	3583 ± 389	3831 ± 262	3605 ± 289	8	7140 ± 244	5913 ± 1216	7380 ± 1051	6811 ± 939	13.8
10^2^		79 ± 2	81 ± 9	78 ± 7.34	79 ± 6.77	8.6	385 ± 104	253 ± 10	253 ± 3.23	297 ± 60	20.2	339 ± 34	334 ± 14	333 ± 23	335 ± 25	7.5
10^3^		7.19 ± 1.77	7.03 ± 2.41	8.49 ± 3.45	7.57 ± 2.63	34.8	23 ± 1.29	20 ± 0.69	22 ± 2.08	22 ± 1.47	6.7	39 ± 15	30 ± 9.18	26 ± 0.83	31 ± 10	32.1
10^4^		0.36 ± 0.62*	0.49 ± 0.24	0.35 ± 0.38^†^	0.4 ± 0.45	111.3	1.04 ± 0.39	0.73 ± 0.26	1.16 ± 0.94	0.98 ± 0.61	62.1	1.65 ± 0.29	1.71 ± 0.32	2.25 ± 0.06	1.87 ± 0.25	13.5
10^5^		0	0	0.17 ± 0.3*	0.06	300	0.17 ± 0.15^†^	0.16 ± 0.14^†^	0.16 ± 0.14^†^	0.16 ± 0.14	86.9	0.44 ± 0.41^†^	0.41 ± 0.49^†^	0.53 ± 0.54^†^	0.46 ± 0.48	104.5

(a) Each run represents the mean Ct value of three replicates within an independent run.

*Only one of three replicates were positive.

^†^Only two of three replicates were positive.

The ‘U’ indicates underdetermined.

(b) Each run represents the mean copy number of three replicates within an independent run.

### LoD assessment using cultured and mock clinical RNAs

The LoD of both the RT-qPCR and RT-ddPCR was determined using serially diluted low concentration viral and mock clinical RNAs. The concentration of the undiluted cultured viral RNA was initially measured using RT-ddPCR, and the concentrations of the L, M, and S segments were 6.84, 1.38, and 2.52 copies/µL, respectively. The concentration of the undiluted mock clinical RNA was also measured using RT-ddPCR, and the concentrations of the L, M, and S segments were 7.57, 0.98, and 1.87 copies/µL, respectively. The measured Ct value and copy number of the serially diluted samples (dilution rate: 2^1^, 2^2^, 2^3^, 2^4^ and 2^5^) are shown in Tables 3 and 4. The LoD of both assays was further investigated with 2^3^ and 2^4^ diluted templates of the viral and mock clinical RNAs in 20 replicates (Table 4). The results show that the RT-ddPCR assays were more sensitive compared with the RT-qPCR assays in terms of the LoD. One exception was the diluted (2^4^) cultured viral RNA. However, the Ct values of these diluted cultured viral RNAs were very high, and most of these Ct values can be regarded as negative, indicating the RT-ddPCR assays were also more sensitive with these samples in practical terms. The overall detection rate of the RT-ddPCR was higher than that of RT-qPCR. Because the high Ct values (more than 37) were generally considered as negative, the LoD of the RT-ddPCR was superior to that of the RT-qPCR.

**Table 3:** Limit of Detection (LoD) of the cultured RVFV RNA by RT-qPCR and RT-ddPCR with the L, M and S segment assay.

Dilution rate	Primer-probes	(a) Cultured RNA					
		RVFV L		RVFV M		RVFV S	
	Platforms	Q	D	Q	D	Q	D
NTC		U	0	U	0	U	0
2^1^	Run1	34.17 ± 0.42	2.88 ± 0.95	38.89 ± 0.72	0.57 ± 0.29	36.71 ± 0.37	1.95 ± 0.65
	Run2	34.72 ± 0.28	2.6 ± 0.98	38.33 ± 0.63^†^	1.15 ± 0.17	37.61 ± 0.94	1.44 ± 1.27
	Run3	34.34 ± 0.5	3.59 ± 1.59	38.05 ± 0.23	0.99 ± 0.5	36.93 ± 0.43	1.11 ± 0.25
	Mean	34.41 ± 0.41	3.02 ± 1.21	38.42 ± 0.57	0.9 ± 0.35	37.08 ± 0.63	1.5 ± 0.84
	RSD (%)	1.2	40.1	1.5	38.5	1.7	55.9
2^2^	Run1	35.6 ± 0.7	0.96 ± 0	39.68*	0.32 ± 0.14	37.78 ± 0.63^†^	0.4 ± 0.37^†^
	Run2	36.14 ± 1.2	1.53 ± 0.43	U	0.25*	38.43 ± 1.35	0.87 ± 0.59
	Run3	35.69 ± 0.51	1.23 ± 0.74	U	0.48 ± 0.42^†^	38.67 ± 0.2^†^	0.4 ± 0.14
	Mean	35.81 ± 0.85	1.24 ± 0.49	39.68*	0.35 ± 0.36	38.29 ± 0.87	0.56 ± 0.41
	RSD (%)	2.4	39.8	0	102	2.3	73.2
2^5^	Run1	38.09 ± 0.76^†^	0.16*	U	0.08*	39.41 ± 0.4^†^	0.08*
	Run2	37.41*	0.16 ± 0.14^†^	39.74*	0.24 ± 0.24^†^	38.25 ± 0.2^†^	0.09*
	Run3	37.21 ± 0.01^†^	0	U	0.08*	39.93*	0.08*
	Mean	37.57 ± 0.44	0.11 ± 0.18	39.74*	0.13 ± 0.18	39.19 ± 0.26	0.08 ± 0.15
	RSD (%)	1.2	167.7	0	134.2	0.7	173.7

Dilution rate	Primer-probes	(b) Mock clinical RNA					
		RVFV L		RVFV M		RVFV S	
	Platforms	Q	D	Q	D	Q	D
NTC		U	0	U	0	U	0
2^1^	Run1	33.73 ± 0.1	1.87 ± 0.68	38.19 ± 1.02	0.6 ± 0.14	36.98 ± 1.24	0.68 ± 0.28
	Run2	33.96 ± 0.45	3.12 ± 1.36	38.16 ± 0.99	0.65 ± 0.34	36.04 ± 0.65	0.67 ± 0.4
	Run3	34.23 ± 0.37	2.61 ± 1.05	37.26 ± 0.85	0.4 ± 0.37^†^	35.92 ± 0.37	0.89 ± 0.61
	Mean	33.97 ± 0.34	2.53 ± 1.07	37.87 ± 0.96	0.55 ± 0.3	36.31 ± 0.83	0.75 ± 0.45
	RSD (%)	1	44.2	2.5	54.7	2.3	60.3
2^2^	Run1	35.36 ± 0.24	1.25 ± 0.47	39.25 ± 0.3	0.69 ± 0.59	37.3 ± 1.3^†^	0.67 ± 0.16
	Run2	35.14 ± 0.22	1.28 ± 0.47	37.86 ± 0.01^†^	0.27 ± 0.02	36.97 ± 0.49^†^	0.41 ± 0.3
	Run3	35.05 ± 0.46	1.07 ± 0.57	38.04 ± 0.85	0.08*	37.84 ± 0.4	0.43 ± 0.16
	Mean	35.18 ± 0.32	1.2 ± 0.51	38.38 ± 0.52	0.35 ± 0.35	37.37 ± 0.84	0.5 ± 0.22
	RSD (%)	0.9	42.1	1.4	101	2.2	43.4
2^5^	Run1	37.28 ± 0.72^†^	0	U	0	37.65 ± 0.8^†^	0.08*
	Run2	37.26 ± 0.63^†^	0.08*	U	0	36.39*	0.24 ± 0.24^†^
	Run3	38.52 ± 0.91^†^	0.08*	U	0	U	0.09*
	Mean	37.69 ± 0.76	0.05 ± 0.11	U	0	37.02	0.14 ± 0.19
	RSD (%)	2	212.1	0	0	0	134.4

Each run represents the mean of three replicates within an independent run.

*Only one of three replicates were positive.

^†^Only two of three replicates were positive.

The ‘U’ indicates underdetermined.

The ‘Q’ indicates RT-qPCR, and the unit is ‘Ct value’.

The ‘D’ indicates RT-ddPCR, and the unit is ‘copy/μl’.

**Table 4:** Comparison of each segment assay on both the RT-qPCR and RT-ddPCR platforms. Analyses of the diluted (2^3^ and 2^4^) cultured and mock clinical RNAs were performed in 20 replicates.

Replicate	Primer-probes	Cultured RNA											
		RVFV L				RVFV M				RVFV S			
	Dilution rate	2^3^		2^4^		2^3^		2^4^		2^3^		2^4^	
	Platforms	Q	D	Q	D	Q	D	Q	D	Q	D	Q	D
NTC		U	0	U	0	U	0	U	0	U	0	U	0
1		37.4	0.24	38	0	U	0	39	0	38	0	U	0.24
2		37.3	0.48	36.8	0.76	U	0	U	0.28	38.4	1.28	U	0.24
3		35.6	0.24	36.4	0.48	U	0.24	U	0	39.5	0.76	39.6	0.24
4		36.9	0.24	U	0.52	U	0.44	U	0	37.4	0.52	U	0.24
5		36.8	1.04	U	0	U	0	U	0	39.4	0.28	39.6	0.24
6		37	0.72	36.2	0	U	0.24	U	0	37.4	0.52	40	0.2
7		35.9	1	U	0.24	U	0	U	0	39.9	0.24	U	0.24
8		36	0.76	38	0	39.9	0.24	U	0	U	0.24	U	0.72
9		35.9	0.24	36.9	0.88	U	0	U	0	38.1	0	U	0.48
10		36	0.8	36.2	0.96	U	1.12	U	0	U	0.24	U	0.24
11		38.1	0.52	38.3	0	U	0	U	0	38.8	0	U	0.24
12		36	0.24	U	0.72	U	0.28	U	0.56	U	1	39	0
13		36.4	1.2	37	0.24	39.6	0	U	0.24	U	0	38.4	0
14		37	1.56	38.5	0.24	U	0.52	39.5	0	39.6	1.04	38	0
15		35.7	0.24	U	0	U	0	U	0	U	0.28	39.7	0.24
16		38.2	0.28	35.9	0.28	U	0.24	U	1.24	39	1	39.5	0.24
17		35.8	0.92	36.4	0.24	U	0	U	0.28	U	0.48	U	0.24
18		36.5	1.16	38	0	39	0.48	U	0.24	39.3	0.24	U	0.76
19		U	1.24	36.1	0	39.1	0.72	U	0.48	39.4	0.24	39.5	0
20		36.2	0.96	37.9	0	U	0.48	39.6	0	38	0	U	0.24
Average		39.6	0.7	37.1	0.28	39.4	0.25	39.4	0.17	38.7	0.42	39.3	0.25
Standard deviation (±)		0.8	0.42	0.9	0.33	0.4	0.3	0.31	0.31	0.83	0.4	0.64	0.2
Detection Rate (%)		95	100	75	55	20	55	15	35	70	75	45	80

Replicate	Primer-probes	Mock clinical RNA											
		RVFV L				RVFV M				RVFV S			
	Dilution rate	2^3^		2^4^		2^3^		2^4^		2^3^		2^4^	
	Platforms	Q	D	Q	D	Q	D	Q	D	Q	D	Q	D
NTC		U	0	U	0	U	0	U	0	U	0	U	0
1		36.9	0.72	35.4	0.24	U	0.28	U	0	38.4	1.20	U	0
2		36.8	0.72	36.4	0	U	0.24	U	0	37.4	0	38.7	0
3		36.3	0	U	0.24	U	0.64	U	0	37.8	0.24	U	0.64
4		37.9	0	36.4	0.24	U	0.48	U	0	U	0.24	37.6	0.52
5		36.8	0.76	37.3	0.48	U	0.28	U	0	36.7	0.84	U	0
6		37.9	0.28	37.1	0.44	U	0.28	U	0.24	37.6	0.52	35.3	0
7		35.3	1	U	0	U	0.56	39.8	0	39.7	0.28	38.8	0.28
8		37.4	0.72	U	0	39.5	0.52	U	0.24	36.8	0.76	U	0.28
9		35.1	1	37.4	0.28	U	0.24	39.8	0	38.0	0	U	1.4
10		37.4	0.52	U	0.24	39.9	0.24	39.7	0	U	0.24	U	1.96
11		37.5	0.24	37.3	0.48	39.9	0.28	U	0.24	38.0	0.24	39.7	0
12		37.3	1	U	0	U	0.24	U	0	36.3	0	U	0
13		35.9	0.52	U	0.24	U	0	U	0	37.7	0	38.4	0
14		36.4	1	U	0.24	U	0.52	U	0	35.9	0.24	37.8	0.48
15		36.5	1.56	37.5	0.76	U	0.24	U	0	37.6	1.12	37.9	0.48
16		35.5	1.24	36.4	0.24	U	0.24	U	5.2	36.0	0	37.9	0.24
17		35.5	1.76	36.8	0	U	1.32	38.7	2.48	37.1	1	U	0.52
18		35.8	0.44	U	0	U	0	U	0	37.1	0	U	0
19		37.4	1.64	U	0.24	40.0	0	U	0.24	37.6	0	37.6	0.52
20		35.2	0.24	35.4	0.52	38.9	0	U	0	37.9	0.8	U	0
Mean		36.5	0.77	36.7	0.24	39.7	0.33	39.5	0.43	37.4	0.39	38	0.37
Standard deviation (±)		0.93	0.51	0.76	0.21	0.44	0.3	0.53	1.25	0.9	0.42	1.16	0.51
Detection rate (%)		100	90	55	70	25	80	20	30	90	65	50	55

The ‘U’ indicates underdetermined.

The ‘Q’ indicates RT-qPCR, and the unit is ‘Ct value’.

The ‘D’ indicates RT-ddPCR, and the unit is ‘copy/μl’.

## Discussion

The results of the RT-qPCR and RT-ddPCR using the RVFV L, M, and S primer–probe sets with eleven RNAs from negative reference viruses as templates showed that the primer–probe sets are clearly specific to RVFV. These results indicate that the primer–probe sets do not react with other mosquito-borne flaviviruses in both RT-qPCR and RT-ddPCR^32–34^. In our previous study, the ddPCR result of a SARS-CoV-2 negative patient with the 2019-nCoV_N2 primer–probe sets was less than 4.3 copies/μl while the result with the WH-NIC N primer–probe sets was less than 0.5 copies/μl^17^. These results indicate that the RVFV primer–probe sets can be very specific and sensitive to the three RVFV strains due to the low background noise. The low level of background noise for RT-ddPCR can be shown according to the characteristics of the primer–probe sets. Different levels of background noises were observed with various SARS-CoV-2 primer–probe sets in a previous study; the ddPCR result of a SARS-CoV-2 negative patient with the 2019-nCoV_N2 primer–probe sets was less than 4.3 copies/μl while the results with the WH-NIC N primer–probe sets were less than 0.5 copies/μl.

The detection and quantification of RVFV IVT RNA using RT-qPCR and RT-ddPCR were compared, and the results with the synthetic RNAs of the three strains in three different lineages proved that the synthetic RVFV RNAs worked well with the previously established RT-qPCR and RT-ddPCR. The primer–probe sets used in this study originally targeted RVFV AR20368 (L, S) and RVFV 35/74 (M) which belong to the C and D lineage, respectively^15,16,35,36^. The cultured viral RNA of the Lunyo strain belongs to the E lineage and also worked well with both assays in this study^35,37^. These results indicate that the primer-probes sets in this study worked well with the different lineages of RVFV. However, the sequences of the primer–probe set for the S segment were identical to only a few numbers of genome sequences and potentially require the development of universal primer–probe sets with more conservative regions. However, the primer–probe set for the S segment worked well with these synthetic RNAs and cultured viral RNAs.

The LoQs of both assays were similar. Due to the principle of ddPCR, the method can detect a single molecule theoretically. The linear regression of the results from both assays showed that the standard curves were linear to a very low concentration. The RSD of RT-ddPCR was approximately 10% except for a very low concentration. Considering the logarithmic property of Ct, the RT-ddPCR assays were more precise than those of the RT-qPCR assays. At a very low concentration (< 10 copy/μl), the standard deviation of most runs was less than 2 copies/μl, indicating the assays can quantify single-molecule level target genes in the samples.

The LoDs of the RT-ddPCR assays were generally superior to those of the RT-qPCR assays. Although both assays showed positive results with a template of less than one copy/μl in the samples, the detection rate of RT-ddPCR was generally higher. Although the detection rate of RT-qPCR was higher in some runs, the majority of the Ct values in these runs were higher than 37 which were generally regarded as negative criteria for qPCR. Because the background signals of qPCR can be influenced by various factors such as reagents, instruments, polymerase, etc., the higher Ct values were not regarded as positive signals. The detection rates of all RT-ddPCR runs were higher than those of RT-qPCR when Ct values more than 37 were regarded as negative signals.

In this study, PCR-based assays for RVFV were assessed using synthetic and cultured RVFV viral RNAs. Because the primer-probes used in this study were designed and validated with the cultured RVFV samples in a previous study, the results of this study suggest that the primer-probes can be used with both RT-qPCR and RT-ddPCR for the detection and measurement of various RVFV variants clinically^15,16^.

Although actual clinical samples were not used in this study, clinical samples were mimicked by using Human Adult Normal Tissue RNA as a matrix. Because the host RNA (human RNA) is expected to be the major non-target RNA of the extracted clinical samples, the results with the mock clinical samples suggest that the assays can produce similar results with actual clinical samples.

These results were also consistent with previous studies. Recently, various RT-qPCR assays for SARS-CoV-2 have been developed and compared using both RT-qPCR and RT-ddPCR^17–19,38^. The Ct values of various primer–probe sets were greatly different in the RT-qPCR assay, but the measured copy numbers were relatively similar^17,19^. These results indicate that RT-ddPCR can be more sensitive compared to RT-qPCR assays. Although the LoQ of the well-optimized RT-qPCR assays can reach that of RT-ddPCR, the Ct values of such low concentration temples can be close to or beyond the cutoff values of the RT-qPCR assays. The results of the RT-qPCR and RT-ddPCR assays also showed that both methods were reliable and concordant, indicating the materials measured by RT-ddPCR can be used as reference materials for RT-qPCR^17,19^. This concordance was also confirmed with DENV^39^ and plasma samples containing SARS-CoV-2^40^. Some studies reported that RT-ddPCR was more sensitive than that of RT-qPCR^41^, and our results show that RR-ddPCR can be more sensitive and precise in terms of the LoD and LoQ.

In conclusion, the RVFV primer–probe sets for the RT-qPCR assays were compatible with the RT-ddPCR assays, and the sensitivity of RT-ddPCR assays were more superior to that of the RT-qPCR assays when considering the cutoff values of the RT-qPCR assays. Moreover, the primer–probe sets used in this study can cover various lineages of RVFV with both the RT-qPCR and RT-ddPCR assays. Because the primer–probe sets are compatible and absolute quantification can be done with RT-ddPCR, the reference materials for RT-qPCR can be measured with RT-ddPCR.

## Materials and methods

### Primer and probes

The forward and reverse primers were synthesized for the amplification of the RVFV genomic segments by Macrogen (Korea). The primer–probe sets of RVFV for RT-qPCR and RT-ddPCR were synthesized by IDT Korea. The probes for RVFV, Chikungunya virus (CHIKV) and Severe fever with thrombocytopenia syndrome virus (SFTSV) were labelled with fluorescence dye 6-carboxyfluorescein (FAM) at the 5′-end and labelled with a dual quencher; the ZEN quencher was a distance of 9-bp from the fluorescence dye, and lowa Black FQ was at the 3′-end^15,16,42^. The probes for the Zika virus (ZIKV), Dengue virus (DENV), and Japanese encephalitis virus (JEV) were labeled with fluorescence dye 6-carboxyfluorescein (FAM) at the 5′-end and Black Hole Quencher 1 (BHQ-1) at the 3′-end and were synthesized by Macrogen (Korea)^42,43^. All sequence information for the primers and probes is shown in Supplementary Table S6.

The genome sequences of RVFV were retrieved from Virus Pathogen Resources (ViPR), a public pathogen virus database, and aligned for phylogenetic analysis with MEGA7 (version 7.0.21). Combining the results of the primer and probe sites comparison with the various RVFV genomes, our alignment with the phylogenetically distant strains of RVFV ZH548, Kenya 56 and BIME01 showed that these primer–probe sets potentially covered diverse RVFV strains. The genome sequence of the Sandfly fever Naples phlebovirus belonging to the same genus was used as an outgroup in the phylogenetic analysis.

### Viral RNA and mock clinical RNA preparation

The viral RNA of RVFV Lunyo (EVAg 005N-02174) was obtained from the Department of Health: Public Health England—Virology & Pathogenesis group (DH) through European Virus Archive global (EVAg). The RNA was extracted from the supernatant of a viral-infected cell culture. The RVFV mock clinical sample was prepared by mixing viral RNA and human tissue RNA in an RNA storage solution^44^. The mock samples were prepared with the following composition: 25 µL of RVFV viral RNA, 25 µL (200 ng/µL) of total RNA of Human Adult Normal Tissue: Blood Vessel: Vein (BioChain, USA), and 200 µL of the RNA Storage Solution (Invitrogen, USA).

### Preparation of the synthetic RVFV genome

The threes strains (ZH548, Kenya56 and BIME01) of RVFV were selected because they belonged to phylogenetically different lineages (A, D and K)^35,45,46^. The nine genomic DNA segments of the three RVFV strains were synthesized and cloned into the PMA or PMA-RQ plasmid from GeneArt synthesis service (ThermoFisher, USA). The information of the synthetic genes is summarized in Supplementary Table S3. The plasmids with the synthetic RVFV genomes were linearized using the Kpn I enzyme (TaKaRa Bio, Japan). The master mix for the restriction enzyme digestion contained 5 µL of plasmid DNA, 1 µL of Kpn I enzyme, 2 µL of 10× L buffer, and 12 µL of sterile purified water. The mixture was incubated at 37 °C for 1 h. The linearized plasmid was purified using the QIAquick PCR purification kit (Qiagen, USA).

The purified linear plasmids were amplified with the primer pairs for in vitro transcription (IVT) template preparation using TaKaRa Ex Taq^®^ (TaKaRa Bio, Japan) (Supplementary Table S6). The PCR reaction mixture had a volume of 25 µL and consisted of 0.5 µL of TaKaRa Ex Taq (5 U/µl), 2.5 µL of 10× Ex Taq Buffer (Mg^2+^ plus) (20 mM), 4 µL of dNTP mixture (2.5 mM each), 2 µL of purified plasmid template, 1 µL of 10 µM T3 forward primer, 1 µL of 10 µM reverse primer, and 14 µL of nuclease-free water. The PCR was carried out under the following conditions: initial denaturation at 95 °C for 5 min, followed by 25 cycles of denaturation at 95 °C for 30 s, annealing at 60 °C for 30 s and extension at 72 °C (for L, 6 min; for M, 4 min, and for S, 2 min) with a final extension at 72 °C for 5 min. The PCR product was purified using a QIAquick PCR purification kit (Qiagen, USA).

### In vitro transcription and quantification of the RVFV RNA

The purified PCR products were used as templates for the IVT of the RVFV genomic RNA. The IVT was done using the EZ™ High Yield In Vitro Transcription kit (Enzynomics, Korea) according to the manufacturer’s instructions with some modifications. The IVT mixture for the L and M segments had a final volume of 20 µL consisting of 4 µL of 5× transcription buffer, 2 µL of 10× MgCl_2_, 2 µL of DTT (100 mM), 1 µL of 20× Enhancer solution, 2 µL of rATP (100 mM), 2 µL of rGTP (100 mM), 2 µL of rCTP (100 mM), 2 µL of rUTP (100 mM), 1 µL of T3 RNA polymerase, and 2 µL of the template. The mixture for the S segment had a final volume of 20 µL consisting of 4 µL of 5× transcription buffer, 2 µL of 10× MgCl_2_, 2 µL of DTT (100 mM), 1 µL of 20× Enhancer solution, 1 µL of rATP (100 mM), 1 µL of rGTP (100 mM), 1 µL of rCTP (100 mM), 1 µL of rUTP (100 mM), 1 µL of T3 RNA polymerase, 2 µL of the template, and 4 µL of DNase-free water. The mixtures were incubated at 37 °C for 2 h. After incubation, the L, M and S segment mixtures were treated with DNase I (Enzynomics, Korea) to remove any remaining DNA. The mixtures had a final volume of 30 µL consisting of 3 µL of 10× DNase I buffer, 2 µL of Recombinant DNase I (RNase-free), 5 µL of DNase-free water and 20 µL of the in vitro transcription RNA. The mixtures were incubated at 37 °C for 1 h and thereafter purified using RNA Clean & Concentrator-5 (ZymoResearch, USA). The purified IVT RNA was analyzed with the Agilent 2100 Bioanalyzer Instrument (Agilent, USA). The analysis used the Agilent RNA 6000 pico kit (Agilent, USA) after the IVT RNA was serial diluted with RNase-free water. The RVFV IVT RNA was synthesized to cDNA, and gel electrophoresis was performed after the PCR with the primer. The cDNA was synthesized using the IVT RNAs of nine RVFV strains with T3 promoter-tagged primers (Supplementary Table S6). All the PCR products were confirmed with gel electrophoresis, indicating the IVT of the RNA was synthesized completely.

### Negative reference viral RNA genome

The genomic RNAs of nine viruses were obtained from the NCCP (National culture collection for pathogens, Korea) and used as negative references. Supplementary Table S1 shows the information of the eleven negative references. Those viral RNAs were initially quantified using a Quantus fluorometer and serially diluted for the RT-qPCR and RT-ddPCR.

### RT-qPCR of the viral RNA

**Figure 3:**
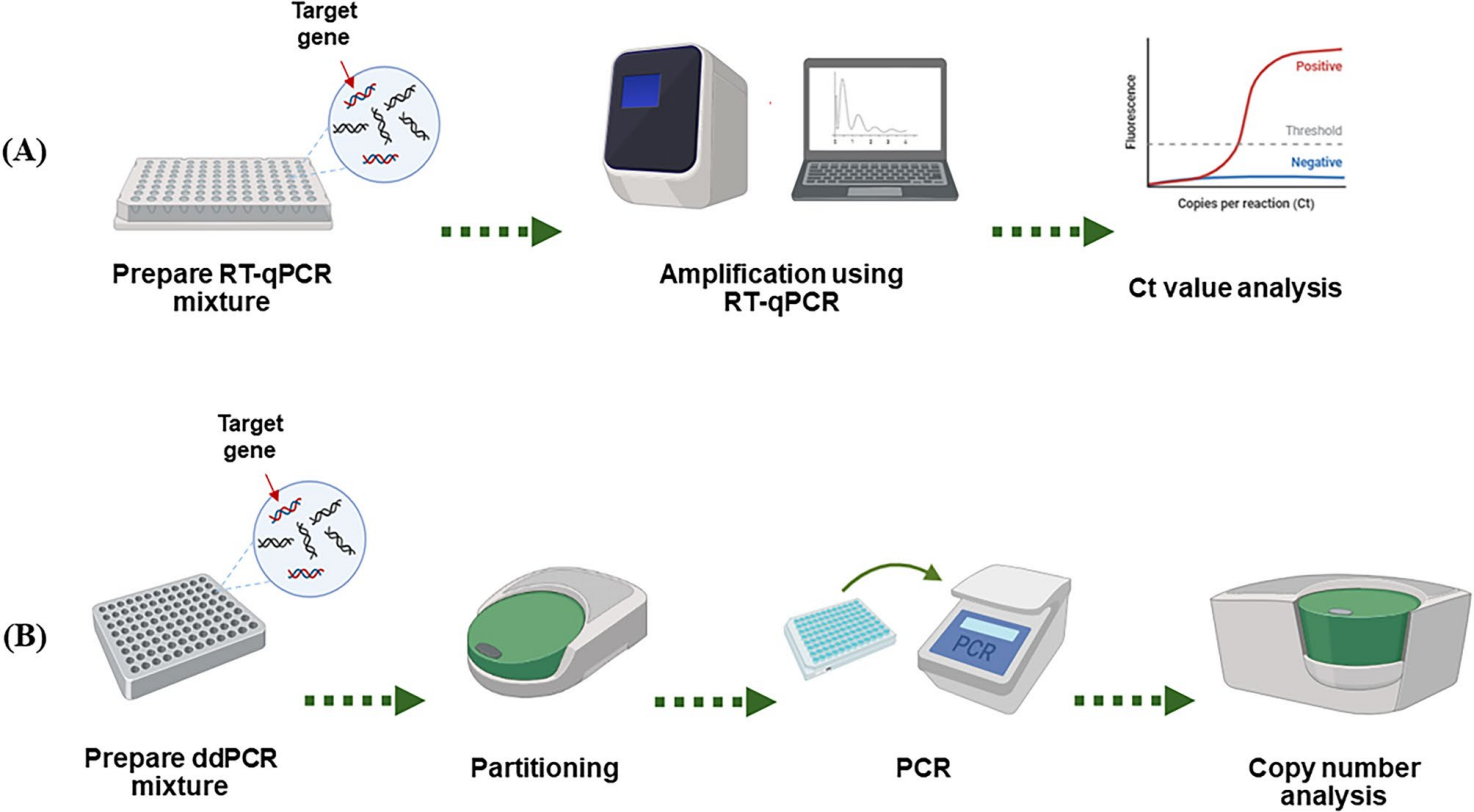
Two PCR-based methods for quantification of the rift valley fever virus (RVFV). (**A**) The Reverse transcription-quantitative polymerase chain reaction (RT-qPCR). (**B**) Reverse transcription-Droplet digital PCR (RT-ddPCR). The schematic figure was drawn using Biorender.

All viral RNAs and synthesized RNAs were used as templates for RT-qPCR using the StepOne and StepOne Plus Real-Time PCR system (ThermoFisher, USA). The RT-qPCR mixtures had a final volume of 20 µL consisting of 10 µL of 2× one-step RT-PCR buffer III (TaKaRa Bio, Japan), 0.4 µL of TaKaRa Ex Taq HS (5 U/µL), 0.4 µL of primescript RT enzyme mix II (TaKaRa Bio, Japan), 0.4 µL of 50× ROX reference dye, 1 µL of 10 µM forward primer, 1 µL of 10 µM reverse primer, 1 µL of a 5 µM probe with 5′-FAM-labeled, 5 µL of RNA template and 0.8 µL of nuclease-free water with one-step PrimeScript™ RT-PCR Kit (TaKaRa Bio, Japan). The RT-qPCR was carried out under the following conditions: reverse transcription at 42 °C for 5 min and enzyme activation at 95 °C for 10 min followed by 40 cycles of denaturation at 95 °C for 10 s and annealing and extension at 60 °C for 35 s (Fig. 3A). In addition, six templates were used for the standard curves except for templates at a number at the attogram level.

### RT-ddPCR of the viral RNA

All viral RNAs and synthesized RNAs were used as templates for the RT-ddPCR assay, and the assay was done with a QX200 system (BioRad, Hercules, CA, USA) and the same primer–probe sets used in the RT-qPCR assay (Supplementary Table S6). The RT-ddPCR mixtures had a final volume of 20 µL consisting of 5 µL of supermix (BioRad, Hercules, CA, USA), 2 µL of reverse transcriptase (BioRad, Hercules, CA, USA), 1 µL of DTT (300 mM) (BioRad, Hercules, CA, USA), 1 µL of 10 µM forward primer, 1 µL of 10 µM reverse primer, 1 µL of 5 µM probe with 5′-FAM-labeled, 5 µL of IVT RNA template and 4 µL of nuclease-free water using a one-step RT-ddPCR advanced kit for the probes (BioRad, Hercules, CA, USA). RT-ddPCR was carried out under the following conditions: reverse transcription at 42 °C for 60 min and enzyme activation at 95 °C for 10 min followed by 70 cycles with a 0.8 °C/s ramp rate of denaturation at 95 °C for 30 s and annealing and extension at 60 °C for 150 s with enzyme deactivation at 98 °C for 10 min (Fig. 3B). The final copy numbers were determined according to the manufacturers’ instructions^20^.

### LoQ (Limit of quantification) and LoD (Limit of detection) of the RT-qPCR and RT-ddPCR

The serially diluted viral and mock clinical RNAs were used to determine the LoD and LoQ of both assays^31^. The expected copy number of those samples was estimated from the measurement of the undiluted samples using RT-ddPCR^20^. The LoQ of both assays was determined using the serially diluted templates (10^0^ to 10^–5^ from original). Three runs were performed for each template, and the measurement of each run was done in triplicate. The standard curves were drawn with the results of the runs without undetermined or undetected results. The LoD of both assays was determined using a serially diluted low concentration RNA (2^1^ to 2^5^ from 10^–4^ or 10^–5^). Three independent runs were performed with triplicate reactions. The pooled standard deviation (PSD) was calculated by the following equation, and the relative standard deviation (RSD) was calculated using PSD.

$${S}_{pooled}=\sqrt{\frac{{S}_{1}^{2}+{S}_{2}^{2}+{S}_{3}^{2}}{3}}.$$

The reactions of both assays were replicated 20 times with two differently diluted low concentration RNAs for the determination of the LoD.

## Supplementary Information

Supplementary Information.

## Data Availability

All data generated or analyzed during this study are included in this published article (and its supplementary information files).
